# Anti-Alzheimer Activity of Combinations of Cocoa with Vinpocetine or Other Nutraceuticals in Rat Model: Modulation of Wnt3/β-Catenin/GSK-3β/Nrf2/HO-1 and PERK/CHOP/Bcl-2 Pathways

**DOI:** 10.3390/pharmaceutics15082063

**Published:** 2023-07-31

**Authors:** Karema Abu-Elfotuh, Amina M. A. Tolba, Furqan H. Hussein, Ahmed M. E. Hamdan, Mohamed A. Rabeh, Saad A. Alshahri, Azza A. Ali, Sarah M. Mosaad, Nihal A. Mahmoud, Magdy Y. Elsaeed, Ranya M. Abdelglil, Rehab R. El-Awady, Eman Reda M. Galal, Mona M. Kamal, Ahmed M. M. Elsisi, Alshaymaa Darwish, Ayah M. H. Gowifel, Yasmen F. Mahran

**Affiliations:** 1 Clinical Pharmacy Department, Faculty of Pharmacy (Girls), Al-Azhar University, Cairo 11651, Egypt; karimasoliman.pharmg@azhar.edu.eg; 2 Anatomy Department, Faculty of Medicine, Girls Branch, Al-Azhar University, Cairo 11651, Egypt; amina.tolba65@gmail.com; 3 College of Dentistry, University of Alkafeel, Najaf 54001, Iraq; furqan.alshwaily@alkafeel.edu.iq; 4 Department of Pharmacy Practice, Faculty of Pharmacy, University of Tabuk, Tabuk 71491, Saudi Arabia; 5 Department of Pharmacognosy, College of Pharmacy, King Khalid University, Abha 62521, Saudi Arabia; salshhri@kku.edu.sa; 6 Pharmacology and Toxicology Department, Faculty of Pharmacy (Girls), Al-Azhar University, Cairo 11651, Egypt; azzamoro@gmail.com (A.A.A.); mona.kamal@hotmail.com (M.M.K.); 7 Research Unit, Egypt Healthcare Authority, Ismailia Branch, Ismailia 41522, Egypt; sarahmmph822@gmail.com; 8 Physiology Department, Faculty of Medicine (Girls), Al-Azhar University, Cairo 11651, Egypt; nehalabdelmonem.medg@azhar.edu.eg; 9 Physiology Department, Faculty of Medicine (Boys), Al-Azhar University, Demietta 34517, Egypt; magdyyoussef11175@domazhermedicine.edu.eg; 10 Department of Anatomy and Embryology, Faculty of Medicine (Girls), Al-Azhar University, Cairo 11651, Egypt; ranonmohammed2009@gmail.com; 11 Biochemistry and Molecular Biology Department, Faculty of Pharmacy (Girls), Al-Azhar University, Cairo 11651, Egypt; r.awady@yahoo.com (R.R.E.-A.); ph.eman.reda@gmail.com (E.R.M.G.); 12 Biochemistry and Molecular Biology Department, Faculty of Pharmacy (Boys), Al-Azhar University, Cairo 11651, Egypt; ahmed.elsisi@nub.edu.eg; 13 Biochemistry Department, Faculty of Pharmacy, Nahda University (NUB), Beni-Suef 62521, Egypt; 14 Biochemistry Department, Faculty of Pharmacy, Sohag University, Sohag 82524, Egypt; alshaymaa.darwish@pharm.sohag.edu.eg; 15 Pharmacology and Toxicology Department, Faculty of Pharmacy, Modern University for Technology and Information (MTI), Cairo 11571, Egypt; ayah.gowifel@pharm.mti.edu.eg; 16 Pharmacology & Toxicology Department, Faculty of Pharmacy, Ain Shams University, Cairo 11566, Egypt; jassie_81@hotmail.com

**Keywords:** Alzheimer’s disease, GSK-3β-Wnt/β-catenin, PERK/CHOP/Bcl-2, oxidative stress, cocoa, vinpocetine

## Abstract

Alzheimer’s disease (AD) is a devastating illness with limited therapeutic interventions. The aim of this study is to investigate the pathophysiological mechanisms underlying AD and explore the potential neuroprotective effects of cocoa, either alone or in combination with other nutraceuticals, in an animal model of aluminum-induced AD. Rats were divided into nine groups: control, aluminum chloride (AlCl_3_) alone, AlCl_3_ with cocoa alone, AlCl_3_ with vinpocetine (VIN), AlCl_3_ with epigallocatechin-3-gallate (EGCG), AlCl_3_ with coenzyme Q10 (CoQ10), AlCl_3_ with wheatgrass (WG), AlCl_3_ with vitamin (Vit) B complex, and AlCl_3_ with a combination of Vit C, Vit E, and selenium (Se). The animals were treated for five weeks, and we assessed behavioral, histopathological, and biochemical changes, focusing on oxidative stress, inflammation, Wnt/GSK-3β/β-catenin signaling, ER stress, autophagy, and apoptosis. AlCl_3_ administration induced oxidative stress, as evidenced by elevated levels of malondialdehyde (MDA) and downregulation of cellular antioxidants (Nrf2, HO-1, SOD, and TAC). AlCl3 also upregulated inflammatory biomarkers (TNF-α and IL-1β) and GSK-3β, leading to increased tau phosphorylation, decreased brain-derived neurotrophic factor (BDNF) expression, and downregulation of the Wnt/β-catenin pathway. Furthermore, AlCl_3_ intensified C/EBP, p-PERK, GRP-78, and CHOP, indicating sustained ER stress, and decreased Beclin-1 and anti-apoptotic B-cell lymphoma 2 (Bcl-2) expressions. These alterations contributed to the observed behavioral and histological changes in the AlCl_3_-induced AD model. Administration of cocoa, either alone or in combination with other nutraceuticals, particularly VIN or EGCG, demonstrated remarkable amelioration of all assessed parameters. The combination of cocoa with nutraceuticals attenuated the AD-mediated deterioration by modulating interrelated pathophysiological pathways, including inflammation, antioxidant responses, GSK-3β-Wnt/β-catenin signaling, ER stress, and apoptosis. These findings provide insights into the intricate pathogenesis of AD and highlight the neuroprotective effects of nutraceuticals through multiple signaling pathways.

## 1. Introduction

Alzheimer’s is a complex neurological disorder that is progressive. Aβ and tau protein tangles are the key features of AD, found mainly in the entorhinal cortex and hippocampus. The accumulation of Aβ results in impaired cell communication, leading to cell apoptosis. Additionally, an imbalance between Aβ production and clearance strongly correlates with the formation of tau protein tangles [1]. Exposure to aluminum metal is considered the most hazardous risk factor for the etiology of AD [2].

Mounting scientific evidence has firmly linked the onset of AD pathology to oxidative stress. Oxidative stress induces both Aβ accumulation and tau protein phosphorylation, contributing to the pathogenesis of AD [1]. Nuclear factor-erythroid 2-related factor 2 (Nrf2) (Nrf2) reduces oxidative stress by controlling antioxidant proteins such as heme oxygenase-1 (HO-1), decreasing inflammation, and stopping Aβ and tau phosphorylation, which enhances cognitive abilities, learning, and memory. Conversely, a reduction in the transcription factor Nrf2 levels has been observed in AD. Oxidative stress can lead to Nrf2 downregulation and neuroinflammation. Oxidative stress, inflammation, and Nrf2 transcriptional regulation are intertwined. Hence, this highlights that Nrf2 has emerged as a promising novel pharmacological target in the management of AD [3].

It has linked oxidative stress to histopathological changes in AD that increase glycogen synthase kinase-3 beta (GSK-3β) activity. The elevation of GSK-3β leads to the upregulation of phosphorylated tau protein and the dysregulation of wingless-related integration site (Wnt)/β-catenin signaling. Wnt/β-catenin signaling regulates neurogenesis, synaptic plasticity, and Aβ-induced apoptosis [4].

Additionally, endoplasmic reticulum (ER) stress plays a significant role in the pathogenesis of neurodegenerative disorders, including AD. It triggered the expression of C/EBP homologous protein (CHOP), a pro-apoptotic protein, leading to neuronal cell death. ER, stress also impaired autophagy by deregulating the autophagy protein Beclin-1 level. Impaired autophagy has also been found to contribute to the pathological buildup of tau deposition in AD. Clearing abnormal protein aggregation is essential to preventing AD because of its ability to cause neuronal degeneration [5]. Modulating Beclin-1 and Bcl-2 connections regulates autophagy [6].

AD poses a daunting challenge as it lacks any effective therapeutic interventions. Medications can reduce symptoms and slow disease progression, but there may be limited effectiveness and possible side effects with long-term use. However, recent research has identified a promising approach for modifying the overall course of AD by using herbal medicine and nutraceuticals. Notably, herbal medicine and nutraceuticals are safe, affordable, and widely accessible. They offer a diverse range of medical benefits, including powerful antioxidant and anti-inflammatory properties and the ability to inhibit cell death. These features provide a firm foundation for neuroprotection, ultimately leading to a decline in AD symptoms and an enhancement in overall quality of life [7].

Cocoa is the mature fruit of the cocoa tree (*Theobroma cacao L*.), which possesses potent antioxidants, anti-inflammatory, anti-proliferative, and neuroprotective activities. Cocoa flavonoids are protective against minor cognitive impairment and dementia in AD [8]. Vinpocetine (VIN) is an artificial derivative of the vinca alkaloid. It improves brain metabolism and elevates cognitive power. Therefore, it is used in stroke and other cerebrovascular disorders [9]. EGCG, the principal component of green tea, has been studied for its ability to treat inflammation and neurodegeneration [10]. It is well known for its ability to scavenge the free radicals and for its antioxidant and anti-apoptotic properties [9]. Wheatgrass (WG), an early grass of the wheat plant (*Triticumaestivum*), also possesses potent antioxidant effects because of its abundant chlorophyll, vitamins (A, C, and E), bioflavonoids, and mineral nutrients [11]. Another naturally occurring molecule that resembles a vitamin is coenzyme Q10 (CoQ10), which controls mitochondrial oxidative phosphorylation and, consequently, ATP synthesis [12]. CoQ10 has a potent protective effect against experimental cerebral ischemia/reperfusion injury [13] and AD [14]. In addition, the vitamin B complex group, a combination of eight water-soluble vitamins, has displayed potent protective effects against neurodegenerative diseases such as AD [15]. Clinically, Vit B complex is used to improve neurodegeneration [16]. Moreover, the combination therapy of vitamin C (Vit C), vitamin E (Vit E), and Selenium (Se) acts synergistically to provide valuable antioxidant protection against free radical-triggered cell membrane lipid peroxidation [17]. It has been previously stated that Se, Vit E, and Vit C activities are interconnected. Furthermore, deficiencies in these nutrients can cause various neurodegenerative diseases [18].

Our study builds upon previous research and aims to unravel the complex pathophysiological mechanisms involved in AD. Specifically, we sought to investigate the potential neuroprotective impact of cocoa, either alone or in combination with other nutraceuticals such as VIN, CoQ10, EGCG, WG, a combination of Vit E, Vit C, and Se, or the Vit B complex, using an animal model of aluminum-induced AD. Unlike prior investigations that only examined isolated compounds without comprehensive evaluations of behavioral and histopathological outcomes, our study provides a more comprehensive understanding of both the potential neuroprotective properties of these compounds and the additive effects that may arise from their combination. By evaluating critical pathways involved in AD, including oxidative stress, antioxidants, inflammation, ER stress, autophagy, Wnt3/β-Catenin/GSK-3β, and apoptosis, we can better grasp the underlying pathogenesis of the disease. Additionally, we explore how these nutraceuticals impact the pathways to yield neuroprotective effects.

Overall, our study offers valuable insights into the multifaceted pathogenesis of AD and highlights the potential of various nutraceuticals to exert neuroprotective effects through multiple signaling pathways. These findings could guide the development of novel therapeutic approaches for the treatment and prevention of AD.

## 2. Materials and Methods

### 2.1. Ethical Approval

The Animal Care and Use committee of the Faculty of Pharmacy, Al-Azhar University, reviewed and accepted the study protocol with ethical approval number 218/2021. The research complies with the ARRIVE criteria and follows the guidelines outlined in the “Guide for Care and Use of Laboratory Animals”, published by the National Institutes of Health (NIH Publications No. 8023, revised 1978).

### 2.2. Materials

#### 2.2.1. Drugs and Chemicals

Aluminum chloride hydrated (product number: 237078), cocoa extract blend (product number: W584649), CoQ10 (product number: C9538), EGCG (product number: PHL89656), Se (product number: GF59784575), and VIN (product number: V6383) were purchased from Sigma Chemical Co. (St. Louis, MO, USA). Wheatgrass powder was provided by Bioglan Super Foods (Surrey, England, UK). Before oral administration, the WG solution was prepared by suspending it in 1% tween in normal saline. The chemical constituents of WG were previously identified and analyzed in our previous work [19]. To prepare VIN, it was dissolved in distilled water. CoQ10 was dissolved in a 1% aqueous solution of Tween 80. Vit B complex, Vit E, and Vit C were obtained from Kahira Pharmaceutical and Chemicals Ind. Co., Cairo, Egypt. Fresh vitamin E was dissolved in corn oil, and vitamin B complex and vitamin C were dissolved in distilled water. All chemicals used were of the best commercially accessible quality.

#### 2.2.2. Animals

Adult male albino rats (*n* = 90) weighing between 320 and 340 g were provided by Nile Co. for Pharmaceuticals and Chemical Industries, Cairo, Egypt. The rats were purchased and accommodated in cages with three to four rats each under standard laboratory conditions (automatically controlled temperature of 25 °C, humidity, ventilation, and 12-h light/dark cycle). One hour preceding each experiment, rats were taken to experimental locations for acclimatization after food and water were removed from their cages. All studies occurred between the hours of 9 a.m. and 2 p.m.

### 2.3. Methods

#### 2.3.1. Experimental Design

The animals were divided into nine groups (*n* = 10) and injected daily with either saline for control (group 1) or AlCl_3_ (70 mg/kg i.p.) for AD model groups for five weeks [20]. The first AD group served as the model control (group 2). While the other groups were administered AlCl_3_ orally with cocoa (24 mg/kg, group 3) [21], either alone or in combination with EGCG (10 mg/kg, i.p.; Group 4) [22], VIN (20 mg/kg, group 5) [23], CoQ10 (200 mg/kg, group 6) [24], and WG (100 mg/kg, group 7) [19]. Vit B complex (0.2 mg/kg, group 8) [25] was also administered, as was a combination (group 9) of Vit E (100 mg/kg) [26], Vit C (400 mg/kg) [27], and Se (1 mg/kg) [26].

All treatments were administered by gastric gavage, except for AlCl_3_ and EGCG. Four behavioral experiments were performed: The Y-maze, conditioned avoidance, Morris water maze, and swimming tests. Rats were sacrificed 24 h following the final test, and the brain tissues were then removed and subjected to ice-cold saline washing.

#### 2.3.2. Behavioral Tests

##### Y Maze Test

After five weeks, the rats were assessed using the Y maze test to measure spatial working memory, evaluating spontaneous alternation behavior expressed as a percentage and calculated as previously described [28]. The Y maze used in this study was made of black wood and comprised three arms (35 cm long, 25 cm high, and 10 cm wide) with an equilateral triangular central area. During an 8-minute session, the rats had unrestricted access to the maze, starting with one arm. They typically alternated visits between the three arms, as they preferred to explore the arm that had not been recently visited. Effective alternation required the rats to use working memory, maintaining a running list of the arms they had most recently visited and updating it frequently. An entry into an arm was considered when the rat’s rear paws were entirely inside the arm. An alteration occurred when the animal selected an arm different from the one it had previously visited. Although returning to the first arm was considered an error, it was, in fact, the correct answer. To determine the percentage of alternation, the total number of arm entries and their order were recorded, with the arms labeled as A, B, or C. Spontaneous alternation behavior was defined as entrance into all three arms on sequential choices. For example, if the rat made subsequent arm entrances A-C-B-C-A-B-C-A-C-A-B-C-A, it would have completed thirteen arm entrances, eight of which were actual alternations. Cognitive behavior and working memory were calculated as follows:

% Alternations = (Number of actual alterations made/Total number of arm entries − 2) × 100.

The number of maximal spontaneous alternation behaviors was the total number of arm entries minus two [29].

##### Conditioned Avoidance Test (CA)

The conditioned avoidance (CA) test was utilized to assess learning and memory after AD induction [30]. Garofalo et al. adopted an adjusted version of the test to evaluate the impact of treatment strategies on learning capacity post-AD induction. The parameters of the CA test were modified, and its application was expanded to assess learning capacity and memory consolidation under highly stressful circumstances.

The device used for the test consists of five interconnected compartments, with four of them equipped with electrified stainless-steel grid floors used to deliver a shock (unconditioned stimulus; 50 volts, 25 pulses/second). The fifth chamber has a glass floor, representing a safe zone. The training involved pairing an auditory stimulus (an electric bell and a conditioned stimulus) for five seconds with an additional 5 s of foot shock. The number of attempts made by each rat to avoid the electric shock and move to the safety area within five seconds of the conditioned stimulus was recorded on the first and second training days, demonstrating their capacity for learning and short-term memory recall.

##### Morris Water Maze Test

Spatial learning and memory were investigated using the Morris water maze test [31]. Tap water was poured into a circular water tank measuring 150 cm in diameter and 60 cm in height to a depth of 30 cm (25 ± 2 °C), and non-toxic white paint was added to make the water translucent. The pool was virtually divided into four equal quadrants (east, west, north, and south). An escape platform measuring 10 cm in diameter was buried 2 cm beneath the water’s surface at a fixed location in the center of one quadrant. During the trial, the platform remained in the same quadrant. A video monitoring camera above the pool captured the rodents’ swimming path. Each rat was placed into the water with its back towards the pool wall from a specific location in each quadrant and allowed to swim to the platform. Four trials were performed in each of the training sessions given to the rats each day for three consecutive days. The animals had a maximum of 60 s to locate the hidden platform before being allowed to rest on it for 20 s before the start of the next trial. If it took more than 60 s to find the platform, the rat was placed gently on it and given 20 s to rest. The escape latency, or the time taken to locate the platform, was noted. On the fourth day, a probe test was conducted by removing the platform and allowing the rats to swim freely for 60 s. The time spent in the designated quadrant was recorded.

##### Swimming Test

The swimming test was conducted using specific and customized methods [32]. The experiment was performed in a glass tank filled with water and maintained at a controlled temperature of 26 ± 2 °C. One end of the glass tank had a ramp, and the swimming activity began from the opposite side. Each rat was positioned in the tank and given three minutes to reach the ramp using its forepaws. Scores were assigned based on the rats’ behavior: rats that reached the ramp directly received a score of 4, rats that deviated to the right or left before reaching the ramp received a score of 3, rats that deviated in both right and left directions before reaching the ramp received a score of 2, and rats that deviated in various directions away from the ramp before reaching it received a score of 1.

#### 2.3.3. Tissue Sampling and Preparation

Rats were euthanized 24 h after the last behavioral test, and their brain tissues were then excised and carefully cleaned in isotonic saline. Four brains per group were fixed in 10% neutral buffered formalin overnight for histopathological investigations. Each of the remaining six brains was divided into two parts. The first part was homogenized instantly to produce a 10% homogenate (*w*/*v*) using an ice-cold medium containing 50 mM Tris-HCl (pH 7.4) and 300 mM sucrose [33]. For biochemical assays, the homogenate was centrifuged at 1800× *g* for 10 min at 4 °C, and the supernatant was then stored at −20 °C. The second part was reserved at −80 °C to be used in real-time PCR analysis.

#### 2.3.4. Histopathological Examination of Brain Tissue

After being fixed in 10% formalin for 24 h, samples of brain tissue were rinsed with water and serially diluted with alcohol to cause dehydration. The specimens were divided into 4 μm thick segments using a microtome after being immersed in paraffin. The tissue samples were then gathered on glass slides, deparaffinized, and stained with hematoxylin and eosin to perform a routine histological inspection under a light microscope [34].

#### 2.3.5. Biochemical Measurements

##### Fluorometric Technique

After the rats were euthanized, levels of brain monoamines were immediately measured, as alterations in the substance’s level might occur in a matter of minutes. Fluorometric assays of dopamine (DA), norepinephrine (NA), and serotonin (5-HT) were estimated in brain tissue homogenate according to the Ciarlone method [35].

##### Colorimetric Technique

The extent of lipid peroxidation in brain homogenate was measured colorimetrically by assessing malondialdehyde (MDA) using the thiobarbituric acid method (Chemie Gmbh, Steinheim, Germany). Nishilimi methods were used to measure the superoxide dismutase (SOD) enzyme activity based on its ability to reduce the nitro blue tetrazolium dye [36]. Lastly, the antioxidants’ reactions with exogenously provided hydrogen peroxide (H_2_O_2_) were used for total antioxidant capacity (TAC) assessment. The residual H_2_O_2_ was estimated colorimetrically by the enzymatic reaction involving the alteration of 3,5-dichloro-2-hydroxybenzene sulphonate to a colored product.

##### ELISA Technique

Levels of Aβ, brain-derived neurotrophic factor (BDNF), 78 KDa glucose-regulated protein (GRP-78), phosphorylated PKR-like ER kinase (p-PERK)—C/EBP homologous protein (p-PERK/CHOP), and Beclin-1 were measured in brain tissue homogenate using ELISA kits (catalog numbers MBS702915, MBS494147, MBS807895, MBS251116, MBS3808179, and MBS901662, respectively) provided by My BioSource, Inc., San Diego, USA. Ray Biotech ELISA kits (product numbers ELR-IL1b and RTA00) were used to estimate interleukin-1β (IL-1β) and tumor necrosis factor alpha (TNF-α) levels in brain tissue homogenate, respectively. Rat β-catenin ELISA Kit (K3383, Biovision Inc.) and Wnt Family Member 3A (Wnt3a) (orb555678, Biorbyt Ltd., Cambridge, UK) were used to assess their brain concentrations according to the manufacturer’s guidelines. ACHE activity was detected by the ELISA kit (MAK119) provided by Sigma-Aldrich Co. (St. Louis, MO, USA). The quantitative sandwich ELISA method was used consistently with the manufacturer’s instructions.

##### Real-Time Quantitative Polymerase Chain Reaction

The mRNA levels of *Nrf2*, *HO-1*, *GSK-3β*, and *Bcl-2* were assessed using real-time quantitative polymerase chain reaction (RT-qPCR) with the Applied Biosystems Step One Plus apparatus. Total RNA was extracted following the manufacturer’s recommendations using the Qiagen tissue extraction kit (Qiagen, Germantown, MD, USA). The isolated mRNA was reverse-transcribed with a sense rapid cDNA synthesis kit (CAT No. BIO-65053) and then amplified using the Maxima SYBR Green qPCR kit (Fermentas, Hanover, MD, USA). The mRNA levels were detected using the ABI Prism 7500 sequence detector system (Applied Biosystems, Foster City, CA, USA). The results were normalized to β-actin expression using the 2^−∆∆CT^ method to calculate the relative expression of the target genes *Nrf2*, *HO-1*, *GSK-3β*, *Bcl-2*, and β-actin. The primer sequences for the PCR amplification are shown in Table 1.

### 2.4. Statistical Analysis

The one-way ANOVA was employed for multiple comparisons, followed by the Tukey-Kramer test for post-hoc analysis. Results are presented as mean ± SEM, with *p* < 0.05 considered statistically significant. Statistical analysis was conducted using GraphPad Prism software (version 8, ISI^®^, San Diego, CA, USA), and the graphs were generated using the same software.

## 3. Results

In this study, we conducted a comprehensive investigation, including seven different normal control groups of rats, each receiving a distinct intervention in addition to the previously studied groups. These interventions involved cocoa alone, a combination of cocoa with EGCG, VIN, WG, CoQ10, and the Vit B complex, or a combination of Vit E, Vit C, and Se. However, despite the variety of interventions, none of the seven groups showed any significant differences in the measured parameters or histopathological findings compared to the normal control group. For the sake of clarity and simplicity in presenting the data, we did not include these findings in the final research paper.

### 3.1. Behavioral Tests

#### 3.1.1. Y-Maze Test (Percent of Spontaneous Alterations; Assessment of Reference Memory)

**Figure 1:**
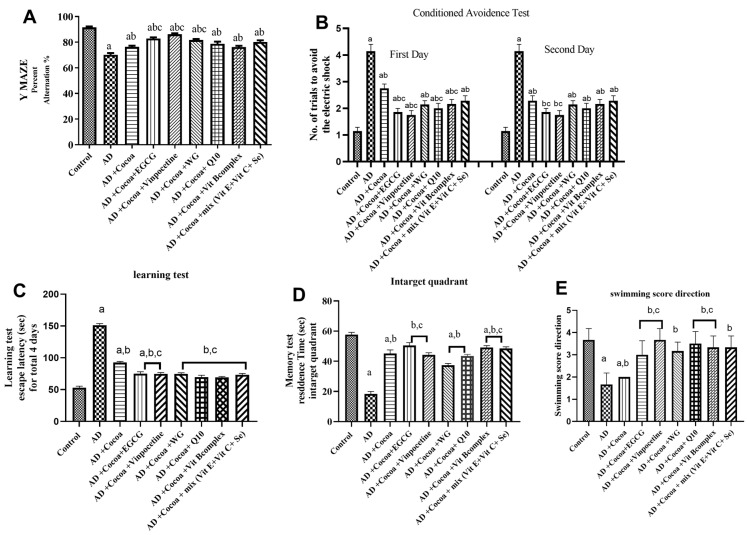
Effect of Cocoa Alone and in Combination with VIN or Other Nutraceuticals on Behavioral Tests in AlCl_3_-induced AD. EGCG; Epigallocatechin-3-gallate, VIN; vinpocetine, WG; Wheatgrass, Q10; coenzyme Q10, Vit; vitamin. (**A**) Effect of treatments on both the locomotor activity and the % spontaneous alternations in the Y-maze model. (**B**) Influence of interventions on the number of attempts to pass the conditioned avoidance test on the first and second days without receiving an electric shock. (**C**) Influence of treatments on the escape latency in Morris water maze test. (**D**) Effect of treatments on the residence time in target quadrant in Morris water maze test (**E**) Effect of treatments on the swimming score direction. Results are established as mean ± SEM, *n* = 6. The significance level at *p* < 0.05). ^a^ indicates significant difference from the control group, ^b^ indicates significant difference from AD group, and ^c^ indicates significant difference from (AD + Cocoa) group. AD; Alzheimer’s disease.

As displayed in Figure 1A, the AD group exhibited a substantial but significant reduction (approximately 23%) in the percentage of continuous alternations when compared to the control group. Treatment with cocoa significantly increased, by approximately 8.5%, the percent of spontaneous alternations when compared to the AD group. Conversely, the influence of the combination of cocoa with VIN, or a mixture of Vit E, C, and Se, revealed the maximum improvement in the percent of spontaneous alterations by 1.2-fold compared to the cocoa-treated group.

#### 3.1.2. Conditioned Avoidance Test (CA) (Assessment of Acquired or Learned Response)

As shown in Figure 1B, the AD group displayed a 5-fold increase on the first day compared to the control group, proving very low short-term memory, and no improvement on the second day relative to the control group. Treatment with cocoa caused a diminution in the number of trials on the first day by 38% related to the AD group. The combination of cocoa and VIN exhibited a maximum further reduction in the number of trials by 65% compared to the cocoa-administered group.

As revealed in Figure 1C, the average latency over the 4 days of training trials increased by 2.2-fold in the AD group compared to the control animals. Management with cocoa resulted in a 34% decrease in escape latency relative to the AD group. The combination of cocoa and either EGCG or VIN exhibited the maximum reversal effect on the spatial memory impairment by 46% and 47%, respectively, relative to the cocoa-treated group.

Figure 1D displayed a 66.5% reduction in the residence time in the AD group relative to the control group, signifying a strong impairment of memory induced by AlCl_3_. The cocoa administration increased the residence time by nearly 2.3 times more than the AD group, which markedly enhanced this subpar performance. The combination of cocoa and either EGCG, VIN, or the mixture of Vit E, Vit C, and Se offered the maximum protection and prolonged residence time (2.8, 2.7, and 2.8-fold, respectively) associated with the cocoa-treated AD group. 3.1.4 Swimming test (ST) (used to reflect cognitive function).

Figure 1E revealed that the AlCl_3_-induced AD group reduced its swimming score direction by 70% compared to the control group. Treatment with cocoa improved the swimming score by 2.6-fold compared with the AD group. Interestingly, co-treatment with cocoa and either EGCG or VIN significantly elevated (3-fold) the swimming score direction compared with the cocoa-treated AD group (adding 60% over the protective effect of cocoa treatment).

### 3.2. Biochemical Measurements

#### 3.2.1. The Effect of Cocoa Alone and in Combination with VIN or Other Nutraceuticals on Oxidative Stress and Antioxidant Biomarkers in Brain Tissues in AlCl_3_-Induced AD

**Figure 2:**
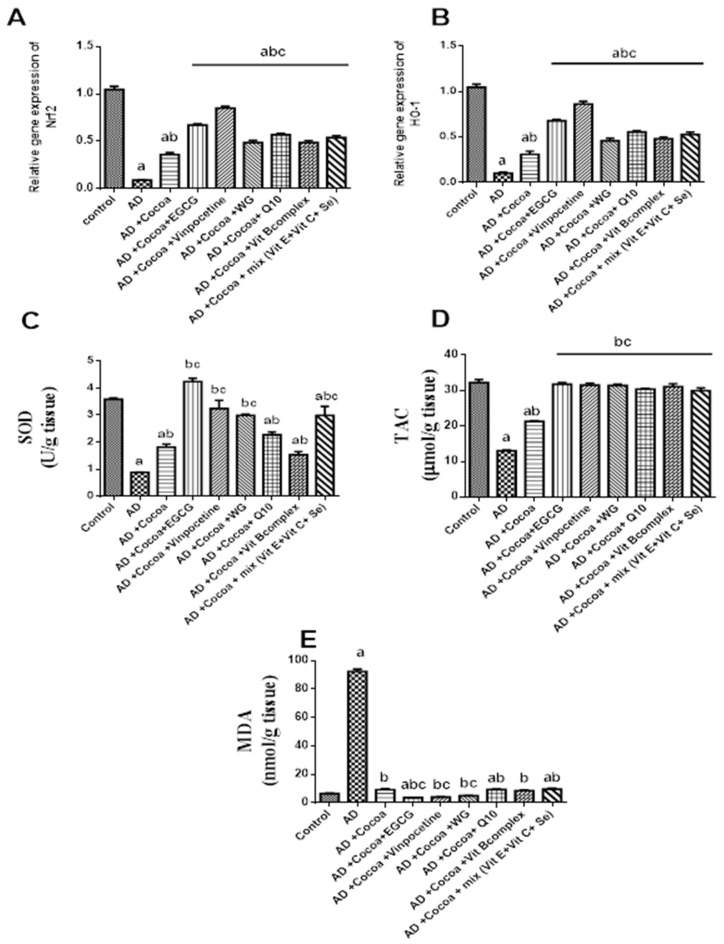
Effect of Cocoa Alone and in Combination with VIN or Other Nutraceuticals on the Oxidative Stress Biomarkers and Antioxidants in Brain Tissue in AlCl_3_-induced AD. EGCG; Epigallocatechin-3-gallate, VIN; vinpocetine, WG; Wheatgrass, Q10; coenzyme Q10, Vit; vitamin. (**A**) Nrf2 gene expression level, (**B**) HO-1 gene expression level, (**C**) SOD level, (**D**) TAC level, and (**E**) MDA level. Results are proved as mean ± SEM, *n* = 6. The significant level at *p* < 0.05. ^a^ indicates significant difference from the control group, ^b^ indicates significant difference from AD group, and ^c^ indicates significant difference from (AD + Cocoa) group. AD: Alzheimer’s disease; Nrf2: erythroid-2 related factor 2; HO-1: Heme oxygenase-1; SOD: Superoxide dismutase; TAC: Total antioxidant capacity; MDA: Malondialdehyde.

As depicted in Figure 2A–C, the administration of AlCl_3_ significantly reduced the mRNA expression levels of antioxidant Nrf2 and HO-1, as well as the activity of SOD, by 91.6%, 90.5%, and 75%, respectively, compared to the control group. In contrast, treatment with cocoa showed a 4-, 3-, and 2-fold increase in the mRNA expression levels of Nrf2 and HO-1 and the SOD activity, respectively, relative to the AD group.

Notably, co-administration of cocoa with either EGCG or VIN resulted in the most substantial elevation in the mRNA expression levels of both Nrf2 (by 7.6- and 9.6-fold) and HO-1 (by 6.6- and 8.4-fold), as well as the activity of SOD (by 4.7- and 3.6-fold), compared to the AD group. Furthermore, the SOD activity in the cocoa combination with EGCG, VIN, and WG did not show significant differences from the normal group.

Figure 2D demonstrates that the AD group exhibited a significantly reduced TAC (total antioxidant capacity) level of 59.6% compared to the control group. However, administration of cocoa mitigated the AlCl_3_ effect, leading to a 1.6-fold increase in TAC level compared to the controls. Co-administration of cocoa with either EGCG or VIN exhibited the highest elevation in TAC level by 2.4-fold compared to the AD group. Additionally, the TAC levels in all cocoa combination groups were not significantly different from the normal controls.

As shown in Figure 2E, the administration of AlCl_3_ resulted in a 14-fold increase in the MDA (malondialdehyde) level compared to the control group. However, cocoa supplementation was able to reverse the AlCl_3_ effects by reducing the MDA level by 90% compared to the control group. Furthermore, co-administration of cocoa with either EGCG, VIN, or WG exhibited a significant maximum reduction in the MDA level by 96%, 95.5%, and 94.4%, respectively, compared to the AD group. The MDA levels also returned to normal in the cocoa treatment and the combination of cocoa with VIN, WG, and Vit B complex.

#### 3.2.2. Effect of Cocoa Alone and in Combination with VIN or Other Nutraceuticals on the Inflammatory Biomarkers in Brain Tissues

**Figure 3:**
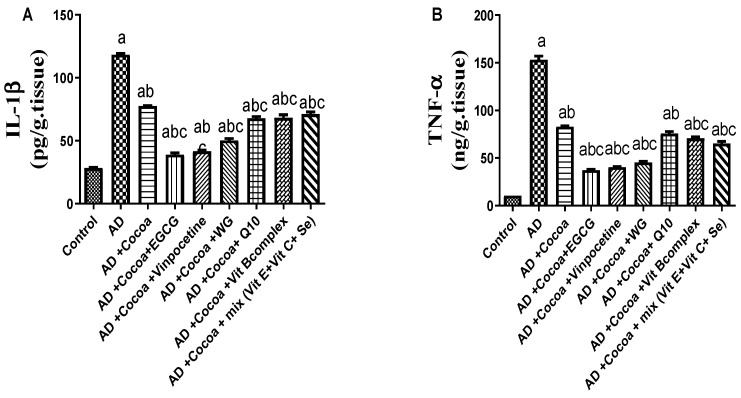
Effect of Cocoa Alone and in Combination with VIN or Other Nutraceuticals on the Brain Inflammatory Mediators. EGCG; Epigallocatechin-3-gallate, VIN; vinpocetine, WG; Wheatgrass, Q10; coenzyme Q10, Vit; vitamin. (**A**) IL-1β level, (**B**) TNF-α level. Results are established as mean ± SEM, *n* = 6. The significant level at *p* < 0.05. ^a^ indicates significant difference from the control group, ^b^ indicates significant difference from AD group, and ^c^ indicates significant difference from (AD + Cocoa) group. AD: Alzheimer’s disease; IL-1β: Interlukin-1β; TNF-α: Tumor necrosis factor alpha.

Figure 3A,B exhibited that the levels of IL-1β and TNF-α were considerably augmented in the brain by 4-fold, 14-fold correspondingly in the AD group versus the control group. Treatment with cocoa diminished the AlCl_3_ mediated inflammatory responses and diminished IL-1β and TNF-α levels significantly by 34% and 46% correspondingly relative to the AD group. Interestingly, co-administration of cocoa with either EGCG, VIN, or WG offered the best downregulation effect on the IL-1β level by 67%, 64.7%, and 57% respectively compared with the AD group. While co-administration of cocoa with either EGCG or VIN could restore the TNF-α brain level and decrease it by 74.5% and 72.8% compared with the AD group.

#### 3.2.3. Effect of Cocoa Alone and in Combination with VIN or Other Nutraceuticals on GSK-3β/BDNF and Wnt/β-catenin Pathways in AlCl_3_-Induced AD

**Figure 4:**
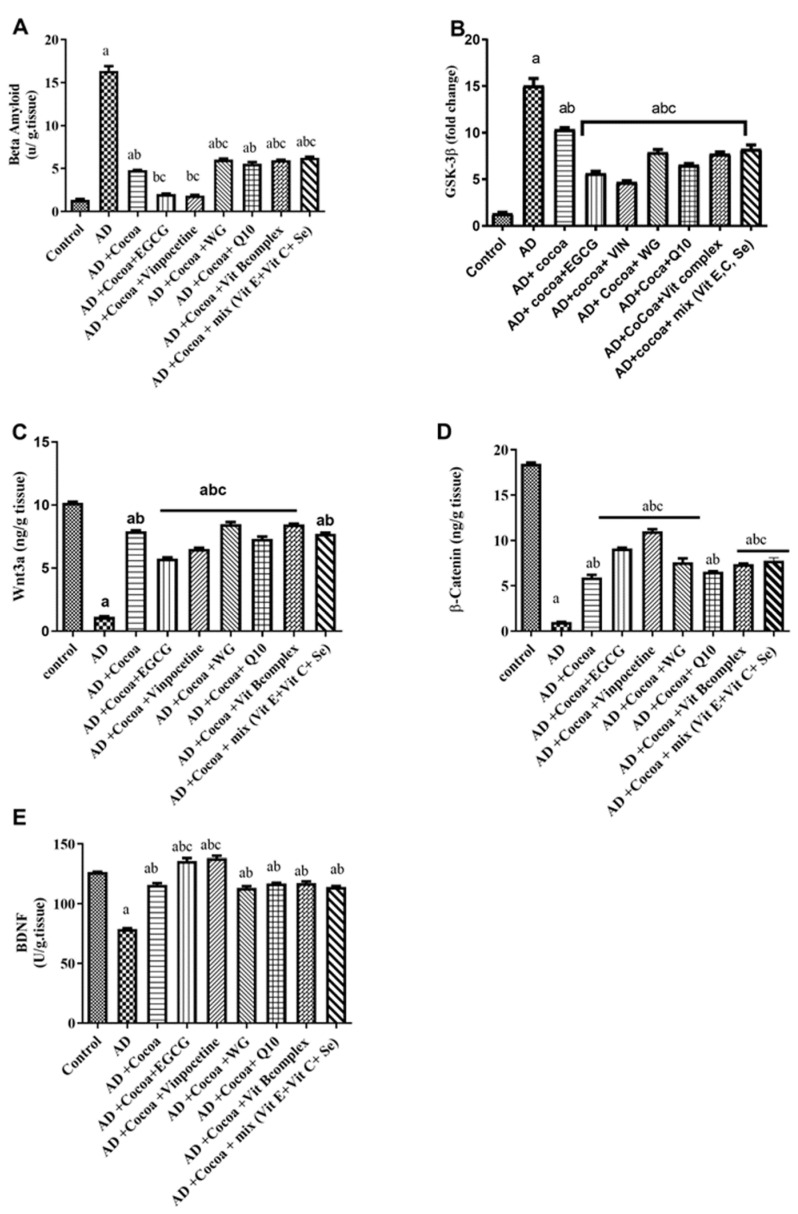
Effect of Cocoa Alone and in Combination with VIN or Other Nutraceuticals on (**A**) Aβ content and (**B**) GSK-3β expression level and (**C**) Wnt3a content (**D**) β-catenin content (**E**) BDNF level in AlCl_3_-induced AD. Results are established as a mean ± SEM, *n* = 6. The significant level at *p* < 0.05. ^a^ indicates significant difference from the control group, ^b^ indicates significant difference from AD group, and ^c^ indicates significant difference from (AD + cocoa) group. AD: Alzheimer’s disease; Aβ: amyloid-beta; Wnt3a: Wnt Family Member 3A; BDNF: Brain-derived neurotrophic factor.

As shown in Figure 4A,B, the AD group exhibited a significant 12-fold and 11-fold increase in Aβ content and GSK-3β expression levels, respectively, compared to the control group. Treatment with cocoa considerably reduced Aβ content by 70.6% and GSK-3β expression level by 31% compared to the AD group. Interestingly, the combinations of cocoa with either EGCG or VIN further reduced the Aβ levels by 87.6% and 88.7%, respectively, compared to the AD group. Moreover, co-treatment with cocoa and either EGCG or VIN maximally decreased the AlCl_3_-induced GSK-3β expression by 62% and 68.8%, respectively.

Figure 4C,D demonstrate that administration of AlCl_3_ resulted in a 7.6-fold and 3.6-fold decrease in Wnt3a and β-catenin levels, respectively, compared to the control group. Treating the rats with cocoa significantly elevated the Wnt3a and β-catenin levels by 20% and 17.5%, respectively, compared to the controls. Co-treatment of cocoa with either EGCG or VIN restored the Wnt3a level in the brain, showing an elevation of 42% and 34%, respectively, compared to the AD group. Similarly, co-treatment with cocoa and either EGCG or VIN augmented the cocoa effect, increasing the β-catenin protein level by 29% and 33%, respectively, compared to the AD group.

As revealed in Figure 4E, the BDNF content was significantly reduced in the AD group by 37.6% compared to the control group. However, treatment with cocoa significantly increased the BDNF content by 1.7-fold compared to the AD group. Interestingly, combinations of cocoa with either EGCG or VIN restored the basal level of BDNF content, showing a 32.8% and 32.7% increase in BDNF content, respectively, compared to the AD group, resulting in maximum cognitive enhancement.

#### 3.2.4. Effect of Cocoa Alone and in Combination with VIN or Other Nutraceuticals on ER Stress, Autophagy, and Apoptotic Markers in AlCl_3_-Induced AD

**Figure 5:**
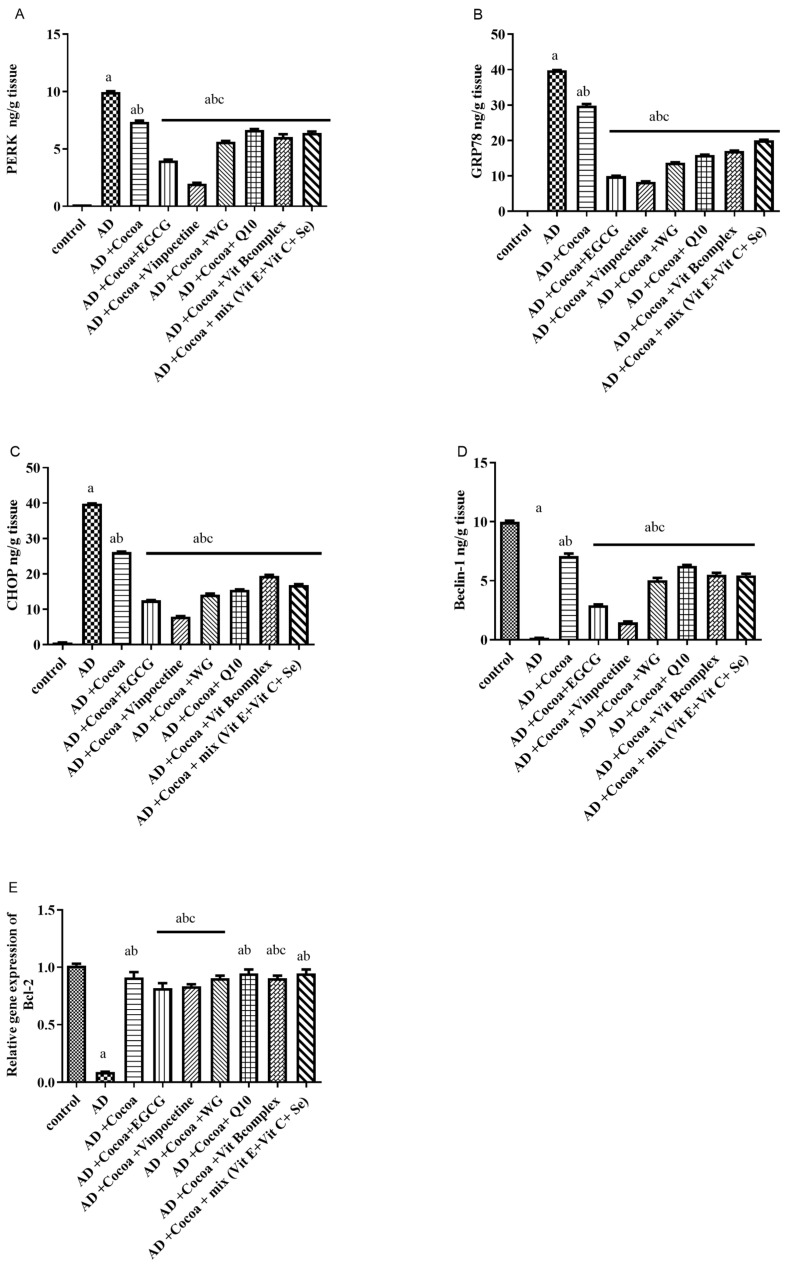
Effect of Cocoa Alone and in Combination with VIN or Other Nutraceuticals on (**A**) p-PERK level (**B**) GRP-78 level (**C**) CHOP level (**D**) Beclin-1 (**E**) *Bcl-2* level, in AlCl_3_-induced AD. EGCG; Epigallocatechin-3-gallate, VIN; vinpocetine, WG; Wheatgrass, Q10; coenzyme Q10, Vit; vitamin. Results are demonstrated as mean ± SEM, *n* = 6. The significant level at *p* < 0.05. ^a^ indicates significant difference from the control group, ^b^ indicates significant difference from AD group, ^c^ indicates significant difference from (AD + Cocoa) group. AD: Alzheimer’s disease; GRP-78: 78 KDa glucose-regulated protein; p-PERK: phosphorylated PERK; CHOP: C/EBP homologous protein.

As shown in Figure 5A–C, there was a significant elevation in the levels of p-PERK, GRP-78, and CHOP by 99-fold, 390-fold, and 66-fold, respectively, in the AD group compared to the control group. Treatment with cocoa decreased the elevated levels of p-PERK, GRP-78, and CHOP by 25%, 25%, and 34%, respectively, compared to the controls. Moreover, co-treatment with cocoa and either EGCG or VIN further augmented the cocoa’s effect, reducing the p-PERK level by 60% and 80%, respectively, compared to the AD group. Additionally, co-administration with cocoa and either EGCG or VIN improved the cocoa’s influence, decreasing the GRP-78 level by 75% and 79%, respectively, relative to the AD group. Furthermore, the co-treatment with cocoa and either EGCG or VIN enhanced the cocoa effect, reducing the CHOP level by 68% and 80%, respectively, compared to the AD group.

Figure 5D,E displayed a substantial decline in the levels of Beclin-1 and the relative gene expression of Bcl-2 by 98% and 92%, respectively, in the AD group compared to the control group. Treatment with cocoa elevated the levels of Beclin-1 and Bcl-2 relative gene expression by 8.9-fold and 9-fold, respectively, compared to the control group. Moreover, co-treatment with cocoa and either EGCG or VIN further augmented the cocoa effect by enhancing the Beclin-1 level by 43-fold and 38-fold, respectively, relative to the AD group. Similarly, co-treatment with cocoa and either EGCG or VIN augmented the cocoa effect and elevated Bcl-2 relative gene expression by 11-fold compared to the AD group.

#### 3.2.5. Effect of Cocoa Alone and in Combination with VIN or Other Nutraceuticals on the Brain Neurotransmitters; Monoamines and ACHE Activity in AlCl_3_-Induced AD

**Figure 6:**
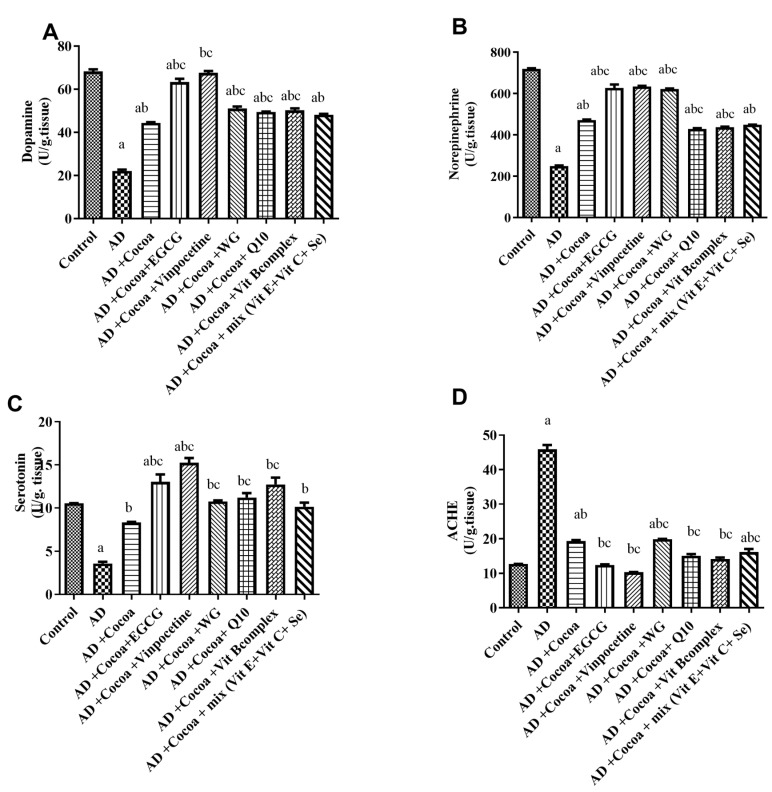
Effect of Cocoa Alone and in Combination with VIN or Other Nutraceuticals on the Brain Monoamine Parameters and ACHE Activity in AlCl_3_-induced AD. Results of (**A**) Dopamine level, (**B**) Norepinephrine level, (**C**) Serotonin level, (**D**) ACHE activity. Results are established as a mean ± SEM, *n* = 6. The significant level at *p* < 0.05. ^a^ indicates significant difference from the control group, ^b^ indicates significant difference from AD group, and ^c^ indicates significant difference from (AD + cocoa) group. AD: Alzheimer’s disease; EGCG: Epigallocatechin-3-gallate; VIN: vinpocetine; WG: Wheatgrass, Q10: coenzyme Q10; Vit: vitamin.

Figure 6A,B show that the AD group had significantly reduced levels of DA and NE by 67.7% and 65%, respectively, compared to the control group. Administration of cocoa caused a 2-fold and 1.8-fold elevation in dopamine and norepinephrine levels, respectively, relative to the AD group. Consistent with previous results, co-administration of cocoa with either EGCG or VIN displayed the highest increase (2.8- and 3-fold rise) in the DA level compared to the AD group. Moreover, co-administration of cocoa with EGCG, VIN, or WG effectively restored the basal level of NE and caused a 2.5-, 2.5-, and 2.4-fold increase in its level, respectively, compared to the AD group.

Figure 6C shows the changes in the cerebral level of serotonin. Treatment with AlCl_3_ revealed a substantial reduction in the level of serotonin by 66% compared to the controls. However, management with cocoa significantly elevated serotonin levels by 2.3-fold compared to the control group. The maximum restoration effect for the serotonin level was observed after treatment with cocoa with either EGCG or VIN, resulting in a 3.6- and 4.2-fold increase in serotonin level, respectively, compared to the AD group.

As displayed in Figure 6D, the administration of AlCl_3_ (70 mg/kg) in the AD group significantly increased the activity of ACHE by 3.7-fold compared to the controls. However, treatment with cocoa decreased the ACHE activity by 57.7% relative to the AD group. Interestingly, co-treatment of cocoa with VIN exhibited a maximum further reduction in the ACHE activity of 77.5% compared to the AD group.

### 3.3. Histopathological Alterations of Brain Tissue in Different Regions

**Figure 7:**
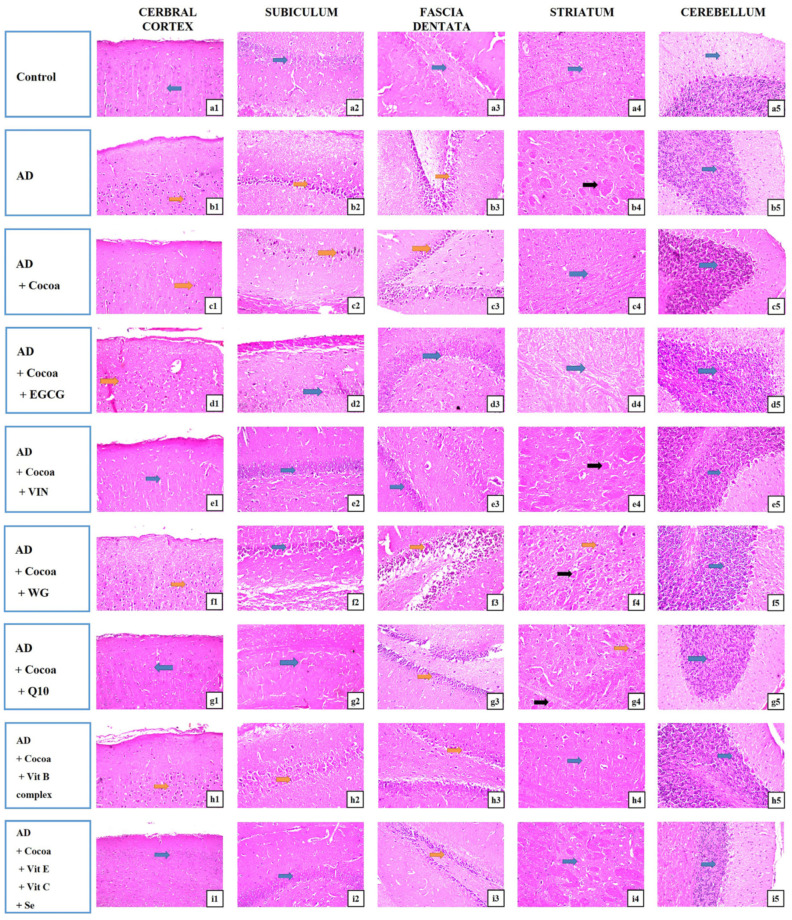
Photomicrographs of brain sections (cerebral cortex, subiculum, and fascia dentata in hippocampus, striatum, and cerebellum areas) stained by hematoxylin and eosin (magnification 40×). AD; Alzheimer disease, EGCG; Epigallocatechin-3-gallate, VIN; vinpocetine, WG; Wheatgrass, Q10; coenzyme Q10, Vit; vitamin. (**a1**–**a5**): control group, (**b1**–**b5**): AD group, (**c1**–**c5**): AD + cocoa group, (**d1**–**d5**): AD+cocoa+EGCG group, (**e1**–**e5**): AD + cocoa + VIN group, (**f1**–**f5**): AD + cocoa + WG group, (**g1**–**g5**): AD + cocoa + CoQ10 group, (**h1**–**h5**): AD + cocoa + Vit B complex group, and (**i1**–**i5**): AD + cocoa + VitE + VitC + Se group. The blue arrow shows no histopathological modification, the orange arrow displays nuclear pyknosis and degeneration, and the black arrow shows focal eosinophilic plagues, the Scare bar was 25µm.

As depicted in Figure 7, the picture of brain tissue segments of rodents stained by H&E stain (magnification 40×) exhibited that in the controls, there was no histopathological change, and the normal histological structure of the neurons was exhibited in the cerebral cortex, subiculum, and fascia dentata in the hippocampus, striatum, and cerebellum regions (Inserts a1, a2, a3, a4, a5). Meanwhile, in the AD group, nuclear pyknosis and degeneration were observed in the cerebral cortex, subiculum, and fascia dentata in the hippocampus, besides multiple large-size focal eosinophilic plagues with damage to the neurons detected in the striatum area. Yet, there was no histopathological modification recorded in cerebellum areas (Inserts b1, b2, b3, b4, b5). While in the AD group treated with cocoa, nuclear pyknosis and degeneration were detected in a few neurons of the cerebral cortex and subiculum and fascia dentata of the hippocampus. In addition, no histopathological change and the normal histological structure of the neurons were established in the striatum and cerebellum (Inserts c1, c2, c3, c4, c5). In the AD group managed with cocoa and EGCG, nuclear pyknosis and deterioration were distinguished in all neurons of the cerebral cortex. There was no histopathological change in the subiculum, fascia dentata, or hilus of the hippocampus, striatum, or the cerebellum (Inserts d1, d2, d3, d4, d5). In the AD group that received cocoa and VIN, there was no histopathological change in the cerebral cortex, subiculum, or fascia dentata of the hippocampus or cerebellum. Focal small-size eosinophilic plagues’ creation with loss in most of the neurons was recorded in the striatum (Inserts e1, e2, e3, e4, e5). In the AD group treated with cocoa and WG, there was no histopathological modification in the hippocampus’s subiculum and cerebellum, but nuclear pyknosis and damage were detected in all the neurons of the cerebral cortex and fascia dentata of the hippocampus. Besides, focal small-size eosinophilic plague formation with nuclear pyknosis in most of the neurons was verified in the striatum area (Inserts f1, f2, f3, f4, f5). In the AD group that received both cocoa and Q10, there was no histopathological modification, as in the cerebral cortex, subiculum of the hippocampus, or cerebellum. Some neurons displayed nuclear pyknosis and degeneration in the fascia dentata of the hippocampus, and there were a few focal eosinophilic small-size plagues produced with nuclear pyknosis in some neurons in the striatum (Inserts g1, g2, g3, g4, g5). In the AD management with both cocoa and the Vit B complex group, there was no histopathological change in the striatum and the cerebellum. However, there was nuclear pyknosis and degeneration in a few neurons of the cerebral cortex, subiculum, and fascia dentata of the hippocampus (Inserts h1, h2, h3, h4, h5). In the AD group administered with cocoa and a mixture of Vit E, Vit C, and Se, there was no histopathological variation in the cerebral cortex, subiculum of the hippocampus, striatum, or cerebellum. Yet, most of the neurons displayed nuclear pyknosis and deterioration in the fascia dentata of the hippocampus (Inserts i1, i2, i3, i4, i5).

As revealed in Table 2, the maximum neuroprotective effect with the least scores was the AD group treated with cocoa either with EGCG or VIN.

## 4. Discussion

AD is a complex neurodegenerative illness characterized by a progressive deterioration in cognitive abilities, including memory, thinking, and learning. Unfortunately, aluminum (Al), which is widely present in our environment and food sources, poses a significant threat to human health and is considered a potential risk factor for AD [2]. As there is currently no definitive therapy for AD, there is an urgent need for innovative treatment strategies that can halt or reverse the devastating effects of the disease. In this context, natural products, especially those derived from plants, offer a promising avenue for developing safe, effective, and affordable therapies for AD. With their unique chemical structures and diverse pharmacological activities, natural products represent a promising frontier in the search for new approaches to AD therapeutics [7]. Thus, our study aims to deeply understand the pathophysiological mechanisms of AD and to assess and compare the protective benefits of cocoa, either individually or in combination with other nutraceuticals. These nutraceuticals have already demonstrated their neuroprotective effects in prior studies or their significance in ameliorating AD symptoms, particularly in AlCl_3_-induced AD. While previous studies have focused on the individual influences of these compounds, our current research aims to provide a more comprehensive understanding of their potential combination of neuroprotective effects.

The outcomes of this study unequivocally establish that chronic daily administration of AlCl_3_ at a dose of 70 mg/kg i.p. for five weeks results in significant neurobehavioral, neurohistopathological, and neurochemical alterations. However, administering cocoa alone or in combination with EGCG, VIN, WG, Q10, the Vit B complex, and a mixture of Vit E, Vit C, and Se was found to be highly effective in providing robust protection against the risks of AD by reversing the adverse effects. These findings have exciting implications for the possible use of these natural compounds in the development of new therapeutic interventions for neurodegenerative illnesses.

In our behavioral study, a significant decline in learning ability and cognitive function was identified, evidenced by an increase in the number of avoidance attempts in the CA test and a decrease in spontaneous alternation in the Y-Maze test among the AD group compared to the control group. These findings suggest a deterioration of learning capability and spatial memory. The declining performance observed in the Morris water maze test supports this. Specifically, the increased escape latency and reduced residence time in the target quadrant among the AD group indicate deficits in learning, memory, and cognitive abilities induced by AlCl_3_ intoxication. Our results are consistent with the prior research conducted by Gu et al. (2009) [37], who also reported significant spatial working memory deficits using the Y-maze among individuals with AD. Additionally, our findings are supported by previous studies that have established behavioral alterations in AlCl_3_-treated rats [38,39]. Administering cocoa alone or in combination with other nutraceuticals resulted in a significant improvement in learning and cognitive function in AlCl_3_-induced AD. In line with our results, previous studies suggested a significant neuromodulator and neuroprotective influence of cocoa flavonoids and their potential for executive ability, behavior, and mental and emotional functions [40]. In addition, the VIN has a neuroprotective influence and can improve learning and memory impairments caused by prolonged cerebral hypoperfusion [9]. EGCG also prevented poor behavioral outcomes associated with AD in rats [19]. Previous studies have revealed that continuous supplementation with Q10, Se, and vitamins (B complex, E, and C) can improve mood and neurotransmitter activity [41].

The observed alterations in behavior in this study were remarkably linked to modifications in underlying histopathological and biochemical parameters. Notably, AD is characterized by severe deterioration of neuronal and synaptic architecture, resulting in the production of Aβ plaques, followed by the buildup of hyperphosphorylated tau protein neurofibrillary tangles in the brain. The extracellular buildup of Aβ and intracellular hyperphosphorylation of the tau protein are the primary culprits of neurons’ degeneration. Soluble Aβ oligomers can also accelerate the onset of tau hyperphosphorylation, leading to impaired plasticity of hippocampal synapses and ultimately causing memory dysfunction (Kitagishi et al., 2014) [42]. The progressive buildup of pathological substances, induced by a complex cascade of events, ultimately results in a critical loss of fundamental cholinergic, synaptic, and cognitive functions, which are the hallmarks of AD [43]. Therefore, understanding the intricate interplay between these processes and identifying effective interventions is a pivotal research pursuit with the potential to improve the devastating impact of these debilitating conditions on affected individuals.

AlCl_3_ induces neuronal oxidative stress [2], resulting in increased expression of free radicals, reactive oxygen species (ROS), and reactive nitrogen species (RNS) [43]. Therefore, oxidative stress triggers destruction in the cellular proteins and nucleic acids, besides lipid peroxidation and raised levels of MDA, a robust biomarker of oxidative stress in the brain [44]. Whereas the Nrf2 transcription factors serve as a critical activators of antioxidant enzymes such as superoxide dismutase-1 (SOD1), HO-1, and CAT to mitigate oxidative stress. Nrf2 also effectively suppresses inflammation mediated by microglia and boosts mitochondrial function. In AD, the Nrf2 pathway undergoes downregulation within the hippocampal neurons because of oxidative stress, leading to a marked reduction in crucial antioxidant enzymes (HO-1, CAT, and SOD1) and a consequent reduction in overall TAC [45,46]. The empirical data corroborate our findings with high consistency and accuracy. However, our drug regimens revealed remarkable antioxidant capabilities, effectively mitigating chronic AlCl_3_-induced oxidative stress. Markedly, co-administration of cocoa with VIN or EGCG emerged as the most efficacious treatment in our study against AD. Cocoa flavonoids are known for their potent antioxidant activity [47]. In the same scenario, VIN and EGCG have antioxidant potential [48,49]. Similarly, Vit B, Se, and Vit E supplementation decreased oxidative stress markers such as MDA [50]. The combination of Vit E and C is valuable and highly synergistic since Vit C can reactivate Vit E back to its reduced form, making it available as an antioxidant again and protecting the membrane from oxidative stress [51].

The accumulation of ROS and concomitant downregulation of Nrf2 play a crucial role in initiating a cascade of inflammation followed by apoptosis, leading to devastating brain injury in AD [52]. TNF-α has been directly linked to Aβ production in AD, while IL-1β has emerged as one of the most prominent cytokines overexpressed during the initial phase of AD pathogenesis [53]. Subsequently, this inflammation contributes to synapse loss, neuronal damage, and AD development [46]. In the present study, our observations have confirmed the activation of pro-inflammatory cytokines, especially TNF-α and IL-1β, in response to the administration of aluminum chloride, inducing the pathogenesis of AD. On the other side, cocoa with VIN or EGCG had the most potent anti-inflammatory effect against AlCl_3_-induced inflammation by reducing TNF-α and IL-1β, among other treatments in this work. Like our results, cocoa exhibited anti-inflammatory activity by reducing inflammatory TNF-α in alcohol-induced liver toxicity models in rats [53]. In addition, it was reported previously that VIN and WG inhibited Aβ-induced toxicity by inhibiting TNF-α and IL-1β [54]. Prior research has revealed that EGCG can mitigate amyloid β-induced toxicity by modulating the activity of TNF-α [55]. Earlier studies exhibited the anti-inflammatory activity of coenzyme Q10 in cerebral ischemia [13], the Vit B complex in wound healing [56], and the combination of Vit E, Vit C, and Se in randomized clinical trials of arthritis [57].

In addition, TNF-α directly contributes to the production of Aβ proteins in AD, which are crucial hallmarks of the disease and play a meaningful role in its progression [52]. In our study, a noticeable increase was observed in the dementia marker Aβ in the AD group, which was consistent with prior research [58]. Combined therapy showed better protection against AlCl_3_ than cocoa alone. This combination resulted in a noteworthy decline in Aβ production. In line with our findings, previous research has shown that cocoa powder administration can reduce Aβ oligomerization [8]. Regarding VIN, its various mechanisms of action are hypothesized to be beneficial in AD [59]. Similarly, EGCG [60] and CoQ10 [61] have been found to reduce Aβ formation in AD transgenic mice. Another study using an in vitro AD model established that Se nanoparticles inhibited Aβ fiber formation [62]. Furthermore, deficiency in vitamins (B complex, E, and C) has been linked to AD patients [63].

The PI3K/AKT/GSK-3β pathway helps promote cell growth and prevent death. This pathway has significant implications for the pathogenesis of various neurological illnesses, including AD. Importantly, it drives the hyperphosphorylation of tau protein, which is a defining hallmark of AD. GSK-3β is the most extensively investigated kinase involved in tau hyperphosphorylation. Furthermore, GSK-3β regulates the neuronal stress response and influences the expression of critical neuropeptides, such as BDNF. These neuropeptides play a vital role in long-term memory and synaptic plasticity. CNS neurons rely on BDNF for survival and differentiation, and its expression is used to measure neurodegenerative changes [64].

GSK-3β is a fascinating multifunctional kinase that is activated by Aβ in AD. It affected multiple signaling pathways, including proinflammatory and proapoptotic [65], and has a critical role in regulating the Wnt/β-catenin pathways. Wnt signaling is an autocrine pathway that has a vital role in brain progress. The elimination of the Wnt3a ligand leads to the disappearance of the hippocampus, underscoring the importance of this pathway in neuronal health [66]. Decreased Wnt activity can intensify the vulnerability of neuronal cells to oxidative insult. Recent research highlights the role of Wnt/β-catenin signaling in inhibiting the production of Aβ and hyperphosphorylation of tau protein in the brain. Consequently, it is involved in the learning and memory processes. Significantly, this pathway is suppressed in the brains of those with AD, pointing towards a potential therapeutic target to mitigate disease progression [66]. GSK-3β is a crucial enzyme responsible for phosphorylating and degrading β-catenin, inhibiting the expression of β-catenin target genes. It has been revealed that the triggering of GSK-3β impedes the Nrf2/HO-1 signaling pathway by augmenting Nrf2 degradation and promoting nuclear factor-κB (NF-kβ) activation, inciting neuroinflammation [67]. Regarding this, it has been established that GSK-3β blockade lowers oxidative injury in a variety of neuronal types [68].

In line with previous findings, our study also revealed the accumulation of Aβ, which triggers the expression of GSK-3β. This leads to the phosphorylation of β-catenin, causing its diminution and the inactivation of Wnt/β-catenin signaling in AD [69]. Consequently, the elevation of GSK-3β reduces the levels of BDNF in the hippocampus, leading to the inhibition of neurogenesis in the brain. Lower BDNF levels have been associated with quicker cognitive decline, poor memory performance, and learning difficulties in AD, as well as other behavioral disturbances in the AD group [70]. However, all our treatment regimens, particularly cocoa alone or in combination with VIN or EGCG, showed improvements in neurogenesis by decreasing GSK-3β activity and consequently activating Wnt/β-catenin signaling, along with increasing BDNF levels. These results align with a previous study that showed cocoa powder’s neuroprotective effects by modulating BDNF in an in vitro human AD model [71]. These results align with an early study that showed cocoa powder’s neuroprotective effects by modulating BDNF in an in vitro human AD model [64]. Similarly, EGCG has been shown to improve functional outcomes after spinal cord injury by targeting BDNF and reducing the level of GSK-3β [72].

Deposition of Aβ and tau proteins triggers ER stress, which can trigger the initiation and progression of the disease [73]. The Unfolded Protein Response (UPR) is a crucial cellular defense mechanism that acts in response to ER stress. PERK regulates the UPR pathway with two other sensor proteins, all of which are inactive under normal GRP-78 conditions. In ER stress, the releasing of GRP-78 triggers the UPR cascade by dimerization and autophosphorylation of PERK and IRE1α. This also leads to the regulated intramembrane proteolysis of activating transcription factor 6 (ATF6). Once active, ATF6 translocated into the nucleus, where it attached to the promoters of many UPR-associated genes, including CHOP [74]. Once activated, CHOP can elicit a cascade of deleterious effects, which can trigger oxidative damage and ROS, augmented levels of Aβ, interference with iron homeostasis, stimulation of inflammation, DNA damage, and ultimately cell death [75].

ER stress increases the level of GSK-3β and subsequently leads to tau phosphorylation. Excessive ER stress can also impair autophagy, which eliminates damaged or misfolded proteins and cellular organelles resulting from oxidative stress [74]. Thus, autophagy plays a crucial role in cell survival regulation. The essential gene Beclin-1 is involved in regulating autophagy and guides the translocation of other autophagy-associated proteins to the autophagosomes. It is vital for neurodegenerative diseases with protein buildup. Depletion of Beclin-1 has been shown to speed up Aβ aggregation and neurodegeneration [76]. A groundbreaking study by Ho and colleagues showed heightened phosphorylation of p-PERK and GRP-78 in the hippocampal region, suggesting continuous ER stress and ineffective UPR. Maladaptive UPR and sustained ER stress can cause impaired autophagy, severe neuroinflammation, and neuron apoptosis, exacerbating the pathophysiology of AD [74]. Elevated GSK-3β and decreased Beclin-1 cause apoptosis and the loss of dopaminergic neurons [74,75]. Additionally, IL-1β has a pivotal role in inducing mitochondrial apoptosis [77]. AD patients with senile plaques in their brains have been observed to exhibit increased caspase activity and changes in levels of apoptosis-related proteins of the Bcl-2 family. Notably, the Bcl-2 protein family serves a crucial function in the intricate interplay between autophagy and apoptosis [75].

In compliance with the previously mentioned mechanism, our results showed an accumulation of Aβ protein leads to ER stress, as proven by elevated levels of ER stress biomarkers, including GRP-78, p-PERK, and CHOP. Consequently, tau phosphorylation increased, elevated GSK-3β levels, and sustained ER stress, which impairs autophagy and reduces Beclin-1 levels. Furthermore, prolonged ER stress induces neural cell death, or apoptosis. The attenuation of the Bcl-2 protein, a key anti-apoptotic regulator, facilitates this process. Additionally, the upregulation of CHOP stimulates apoptosis. In addition, GSK-3β and IL-1β expression cause neural cell death by accelerating apoptosis.

While cocoa alone or in combination with EGCG, VIN, WG, CoQ10, a complex of Vitamin B, or a mixture of Vitamin E, C, and Se exhibited potential for alleviating Al-induced AD, our findings are consistent with preceding research. Cocoa has been shown to improve ER stress levels and prevent apoptosis by elevating the level of anti-apoptotic Bcl-2 [78]. EGCG has diminished ER stress in AD by decreasing GRP-78 and CHOP, regulating autophagy by elevating Beclin-1, and inhibiting neural apoptosis [79]. CoQ10 has also been revealed to improve ER stress, modulate autophagy, and prevent apoptosis [80]. WG can protect against oxidative stress and apoptosis [81]. Vitamin B supplementation has been shown to restore autophagic flux, lessen ER stress, and repair lysosomal dysfunction caused by hyperhomocysteinemia [82]. Besides, it inhibits DNA damage and neural apoptosis [83]. In addition, it diminishes the apoptosis of cells in the rat hippocampus after polyvinyl chloride exposure [84]. Vitamin C is also useful in modulating oxidative stress, autophagy, and apoptosis in bone marrow stromal cells [85]. Finally, selenium deficiency induces inflammation, autophagy, ER stress, apoptosis, and contraction abnormalities by altering the intestinal flora in the intestinal smooth muscle of mice [86]. These results provide a compelling argument for exploring nutritional interventions to combat AD and other disorders associated with ER stress, impaired autophagy, and apoptosis. The central objective of our investigation was to explore the efficacy of nutritional interventions in ameliorating the deleterious effects of AD, with a particular emphasis on the role of cocoa, either alone or in combination with other nutrients, in fostering a potentiating effect that mitigates the advancement of the disease.

Our study revealed a significant reduction in brain monoamines, suggesting neurological damage in AD patients. Previous research has already shown that AD leads to a decline in noradrenergic and serotonergic neurons in the brain, contributing to various behavioral abnormalities [32]. Consistent with these findings, our results show that behavioral disturbances in the AD group are associated with a significant reduction in these monoamines. Notably, our study highlights the potential of cocoa combined with VIN or EGCG to restore reduced monoamine levels effectively, suggesting these nutraceuticals as agents for neuroprotection. Specifically, VIN has been shown to prevent the decrease in the biosynthesis rate of norepinephrine and serotonin [87]. While EGCG [88], Se supplementations, and CoQ10 [89] have been found to prevent the oxidative deamination reaction of amine neurotransmitters. Additionally, previous studies have reported that vitamins C and E can protect against AD in rat models by modulating brain monoamine levels [90]. Furthermore, the observed increase in BDNF levels and decrease in Aβ levels reported in all treatments may be partially attributed to the elevation of brain monoamines [91]. These findings collectively underscore the role of monoamines in apoptosis-related neurological damage in AD. They also suggest that the consumption of cocoa with VIN or EGCG, along with vitamins C and E, holds potential as neuroprotective interventions by enhancing monoamine levels, promoting BDNF synthesis, and reducing Aβ levels.

The investigation found a notable increase in ACHE activity after AlCl_3_ exposure. This observation is in harmony with earlier reports suggesting that Al exposure induces an increase in ACHE activity and consequent pathological deterioration in AD etiology [92]. The capability of Al to perturb the blood-brain barrier and modulate cholinergic neurotransmission is posited as the underlying mechanism for these outcomes. This dysregulation may indicate lysosomal malfunctioning, which could worsen the toxic effects of Aβ [93]. These findings warrant further exploration to unravel the intricacies of the pathophysiology of AD. In the present investigation, all treatments elicited a decline in ACHE activity compared to the AD group, with the combination of cocoa and either VIN or EGCG conferring superior neuroprotection. Data on the effect of cocoa on ACHE is limited, so this study provides the first documentation of cocoa’s potential neuroprotective effects against ACHE in AD. Prior research has established VIN’s ability to attenuate ACHE activity and improve cholinergic function by augmenting acetylcholine levels [9]. Similarly, both EGCG [94] and Q10 [95] administration have been found to mitigate elevated ACHE in streptozotocin-induced dementia models. Moreover, Vit E has been shown to modulate ACHE activity in various brain regions [96]. In the context of Aβ peptide-induced enhancement of ACHE activity, oxidative stress is posited as the underlying mechanism, with Vit E and C successfully abrogating this effect [97,98].

The histopathology analyses confirmed the behavioral and biochemical changes. Nuclear degeneration and pyknosis were found in the cerebral cortex, subiculum, and fascia dentata. These outcomes agree with earlier studies by Abu-Elfotuh et al. and Hamdan et al. [54]. The present study unveils the promising neuroprotective effects of cocoa-based nutraceuticals, either alone or in combination with other dietary supplements, in mitigating the underlying histopathological deterioration. Our study results provide compelling evidence for the potential amelioration effects of the nutraceuticals under investigation, highlighting their therapeutic implications for neurodegenerative disorders. Moreover, our findings suggest that the combination of these compounds potentiates their neuroprotective effects, further emphasizing their potential as a viable treatment option. These promising outcomes pave the way for the development of novel therapeutic strategies that harness the synergistic benefits of these compounds in treating neurodegenerative disorders.

## 5. Conclusions

Our study provides a comprehensive understanding of the complex pathophysiology underlying Alzheimer’s disease, highlighting the interactions between different signaling pathways. By our investigation, we demonstrate the fundamental role of oxidative stress in triggering diminished cellular antioxidants, Aβ and tau protein accumulation, and stimulation of inflammation, sustained ER stress, autophagy, and apoptosis, mediated by pathways such as Wnt/GSK-3β/β-catenin. These alterations lead to neural degeneration, reduced monoamine levels, and changes in brain barrier function and ACHE activity, all of which contribute to the behavioral and histological changes observed in AD.

Our study also highlights the potential of cocoa, either alone or in combination with other nutraceuticals, to ameliorate these biochemical, behavioral, and histological alterations associated with AD, offering a promising avenue for therapeutic intervention to slow cognitive decline. Moreover, the combined intervention of cocoa with VIN or EGCG offers a superior therapeutic effect on behavioral, biochemical, and histological parameters, providing further evidence for the potential of these interventions in the management of Alzheimer’s disease. Further cellular studies are warranted to explore the synergistic effects of these combinations in various experimental systems.

## Figures and Tables

**Table 1:** The sequences of primers employed in real-time RT-PCR analysis.

Gene	Primer Sequence	Accession Number	Product Size (bp)	Annealing Temp. (°C)
*Nrf2*	F: 5′-CTCTCTGGAGACGGCCATGACT-3′ R: 5′-CTGGGCTGGGGACAGTGGTAGT-3′	NM_031789	149 bp	68.4
*HO-1*	F: 5′-CACCAGCCACACAGCACTAC-3′ R: 5′-CACCCACCCCTCAAAAGACA-3′	NM_012580	1043 bp	65.3
*GSK-3β*	F: 5′-AGCCTATATCCATTCCTTGG-3′ R: 5′-CCTCGGACCAGCTGCTTT-3′	NM_032080	701 bp	59.1
*Bcl-2*	F: 5′-GGATGACTTCTCTCGTCGCTAC-3′ R: 5′-TGACATCTCCCTGTTGACGCT-3′	NM_016993	199 bp	64.9
β-actin	F: 5′-CCGTAAAGACCTCTATGCCA-3′ R: 5′-AAGAAAGGGTGTAAAACGCA-3′	NM_031144	299 bp	61.8

**Table 2:** The cerebral cortex and hippocampus histopathological score.

Groups’ Histopathology		Control	AD Group	AD-Treated. with Cocoa	The AD-Induced Group Treated with a Combination of Cocoa with					
					EGCG	VIN	WG	CoQ10	Vit B Complex	Vit E + Vit C + Se
Histopathological Changes	Brain Region									
Nuclear pyknosis and degeneration in the neuronal cells of the of	cerebral cortex	-	+++	+	+	-	+	-	+	-
	the subiculum	-	+++	+	-	-	-	-	+	-
	the fascia dentate of the hippocampus	-	+++	-	-	+	+	+	+	+
Focal eosinophilic plagues in of	the striatum	-	+++	-	-	+	+	+	-	-
Atrophy in the neuronal cells	the cerebellum	-	-	-	-	-	-	-	-	-

AD; Alzheimer disease, EGCG; Epigallocatechin-3-gallate, VIN; vinpocetine, WG; Wheatgrass, Q10; coenzyme Q10, Vit; vitamin. Severe: +++; Mild: +; Nil: -.

## Data Availability

Data will be available on request from the corresponding author.
